# Acknowledgement of manuscript reviewers 2015

**DOI:** 10.1186/s40199-016-0143-z

**Published:** 2016-02-24

**Authors:** Mohammad Abdollahi, Mohammad Ali Faramarzi, Alireza Foroumadi, Kambiz Gilani, Hossein Khalili, Mahnaz Khanavi, Ali Akbar Moghadamnia, Morteza Mahmoudi, Shekoufeh Nikfar, Ali Nokhodchi, Roja Rahimi, Mohammad Reza Rouini, Alireza Vatanara

**Affiliations:** Tehran University of Medical Sciences, Tehran, Iran; Babol University of Medical Sciences, Babol, Iran; University of Sussex, Sussex, UK

## Abstract

The editor of DARU Journal of Pharmaceutical Sciences would like to thank all our reviewers who have contributed to the journal in Volume 23 (2015).

**Akbar Abdollahiasl**

Iran

**Reza Aboofazeli**

Iran

**Hamid-Reza Adhami**

Iran

**Farrokhlagha Ahmadi**

Iran

**Jafar Akbari**

Iran

**Shahin Akhondzadeh**

Iran

**Nafiseh Alizadeh**

Iran

**Reza Alizadeh**

Iran

**Majed Al-Shaeri**

Saudi Arabia

**Mohsen Amin**

Iran

**Kamaran Aryana**

Iran

**Gary Asher**

USA

**Manouchehr Ashrafpour**

Iran

**Abbas Azadmehr**

Iran

**Jonathan Back**

Switzerland

**Jeffrey Brent**

USA

**Abdol Majid Cheraghali**

Iran

**Yung Hyun Choi**

Korea, Republic Of

**Lee Suan Chua**

Malaysia

**Simin Dashti**

Iraq

**Simin Dashti-Khavidaki**

Iran

**Majid Davari**

Iran

**Mouloud Agajani Delavar**

Iran

**Bharat Dixit**

India

**Farid Abedin Dorkoosh**

Iran

**Najmeh Edraki**

Iran

**Joan Estelrich**

Spain

**Anna Maria Fadda**

Italy

**Reza Fardid**

Iran

**Shadi Farsaei**

Iran

**Gholamhossein Farzandy**

Iran

**Omid Firuzi**

Iran

**Hamid Forootanfar**

Iran

**Alireza Foroumadi**

Iran

**Khalil Ghasemi Falavarjani**

Iran

**Kambiz Gilani**

Iran

**Gholamhasan Haddadi**

Iran

**Reza Hajiaghaee**

Iran

**Mehrdad Hamidi**

Iran

**Sharwaree Hardikar**

India

**A Wallace Hayes**

USA

**L Helson**

USA

**Narjes Hendouei**

Iran

**Soheila Hoseinzadeh**

Iran

**Hossein Hosseinzadeh**

Iran

**Fatemeh Imanparast**

Iran

**Hamid Irannejad**

Iran

**Mona Jaberidoost**

Iran

**Bardia Jamali**

Iran

**Zeeshan Javaid**

Pakistan

**Iman Karimzadeh**

Iran

**Hossein Khalili**

Iran

**Mahnaz Khanavi**

Canada

**Mehdi Khoshneviszadeh**

Iran

**Menno LW Knetsch**

Netherlands

**Pravir Kumar**

India

**Shao Lee**

China

**Yu Liu**

China

**Shing Hwa Liu**

Taiwan

**Amelia M Avachat**

India

**Mohammad Mahdavi**

Iran

**Reza Mahjub**

Iran

**Amr Mahmoud**

Egypt

**Morteza Mahmoudi**

Iran

**Hassan Malekinejad**

Iran

**Faheem Maqbool**

Pakistan

**Babak Mirtamizdoust**

Iran

**Sako Mirzaie**

Iran

**Ali Akbar Moghadamnia**

Iran

**Mostafa Mohammadi**

Iran

**Elham Mohit**

Iran

**Sara Mostafalou**

Iran

**Amrollah Mostafazadeh**

Iran

**Stephen A Myers**

Australia

**Hamid Nadri**

Iraq

**Hossein Najafzadeh**

Iran

**Leo Nherera**

UK

**Bahman Nickavar**

Iran

**Wipawee Nittayananta**

Thailand

**N Ohkura**

Japan

**Marcin Ozarowski**

Poland

**Hadi Parsian**

Iran

**Slaven Pikija**

Croatia

**Fereshteh Pourabdolhossein**

Iran

**Ali Pourmand**

USA

**Roja Rahimi**

Iran

**B Renger**

Germany

**Nargess Sadati**

Iran

**Armin Sadighi**

USA

**Soodabeh Saeidnia**

Iran

**Maliheh Safavi**

Iran

**Mohammad Ali Sahraian**

Iran

**Amirhossein Sakhteman**

Iran

**Neda Setayesh**

Iran

**Ahmad Reza Shahverdi**

Iran

**Mojtaba Shakibaie**

Iran

**Hiroshi Shimoda**

Japan

**Esphandiar Shojaei**

Iran

**Khalid Siddiqui**

Saudi Arabia

**Fatemeh Soleymani**

Iran

**Mohammad Soltani**

Iran

**Devendra Swarup**

India

**Maria Tavakoli-Ardakani**

Iran

**Lesheng Teng**

China

**Zahra Tofighi**

Iran

**Maryam Torshabi**

Iran

**Nasir Vadia**

India

**Leila Vali**

Kuwait

**Alireza Vatanara**

Iran

**Mahdi Vazirian**

Iran

**Khushwant S. Yadav**

India

**Narguess Yass**

Iran

**Mohammad Hossein Yazdi**

Iran

**Fatemeh Yousefbeyk**

Iran

**Mir Mahdi Zahedi**

Iran

**Ji Zhang**

China

